# Supplementary material for: Neurotoxin-mediated potent activation of the axon degeneration regulator SARM1
Source: eLife. 2021 Dec 6;10:e72823. doi: 10.7554/eLife.72823 (PMC8758145; doi:10.7554/eLife.72823)
Supplement: Supplementary file 1. [file elife-72823-supp1.docx]

**Supplementary file 1. X-ray data collection and structural refinement statistics.**

| **Data collection** | |
| --- | --- |
| Space group | P1 |
| a, b, c (Å) | 39.06, 51.01, 76.56 |
| α, β, γ (°) | 103.49, 101.78, 96.07 |
| Resolution (Å) | 46.60-1.69 (1.72-1.69) |
| R_merge_ | 0.06 (0.96) |
| R_meas_ | 0.07 (1.13) |
| R_pim_ | 0.04 (0.59) |
| Mean I/σ (I) | 13.7 (1.8) |
| CC_1/2_ | 1.00 (0.69) |
| Total reflections | 428,508 (18,156) |
| Unique reflections | 60,345 (2,695) |
| Completeness (%) | 96.6 (84.6) |
| Multiplicity | 7.1 (6.7) |
| **Refinement** | |
| R_work_ | 0.19 |
| R_free_ | 0.23 |
| RMS bonds (Å) | 0.00 |
| RMS angles (°) | 0.85 |
| Ramachandran favored (%) | 98.68 |
| Ramachandran outliers (%) | 0 |
| Rotamer outliers (%) | 0.19 |
| Clashscore | 2.15 |
| Average B-factor of the protein (Å^2^) | 41.99 |
| Average B-factor of the ligand (Å^2^) | 34.14 |
| C-beta outliers | 0 |

1. The statistics are based on the calculations from Aimless and MolProbity.

2. The numbers in parentheses represent the highest resolution shell.

3. R_merge_ = ∑_hkl_ ∑_j_ |I_hkl,j_ - < I_hkl_ > | / (∑_hkl_∑_j_I_hkl,j_); R_meas_ = ∑_hkl_ [N/(N-1)]^1/2^ ∑_j_ |I_hkl,j_ - < I_hkl_ > | / (∑_hkl_∑_j_I_hkl,j_); R_pim_ = ∑_hkl_ [1/(N-1)]^1/2^ ∑_j_ |I_hkl,j_ - < I_hkl_ > | / (∑_hkl_∑_j_I_hkl,j_)

4. R_work_ = ∑_hkl_ |Fobs_hkl_ - Fcalc_hkl_| / ∑|Fobs_hkl_|; R_free_ is equivalent to R_work_, with 5% of data excluded from refinement process. |Fobs_hkl_| and |Fcalc_hkl_| represent the observed and calculated structure factor amplitudes.
